# Ten-year changes in the prevalence of overweight, obesity and central obesity among the Chinese adults in urban Shanghai, 1998–2007 — comparison of two cross-sectional surveys

**DOI:** 10.1186/1471-2458-13-1064

**Published:** 2013-11-12

**Authors:** Xuhong Hou, Yu Liu, Huijuan Lu, Xiaojing Ma, Cheng Hu, Yuqian Bao, Weiping Jia

**Affiliations:** 1 Department of Endocrinology and Metabolism, Shanghai Jiao Tong University Affiliated Sixth People’s Hospital, Shanghai Clinical Center for Diabetes, Shanghai Diabetes Institute, Shanghai Key Laboratory of Diabetes Mellitus, Shanghai Key Clinical Center for Metabolic Disease, 600 Yishan Road, Shanghai 200233, China

**Keywords:** Central obesity, Chinese adults, Cross-sectional survey, Overweight, Obesity, Prevalence, Urban

## Abstract

**Background:**

In China, obesity is expected to increase rapidly in both urban and rural areas. However, there have been no comprehensive reports on secular trends in obesity prevalence among Chinese adults in urban Shanghai, which is the largest city in southern China.

**Methods:**

In 1998–2001 and again in 2007–2008, two independent population-based cross-sectional surveys were conducted in Shanghai to investigate the prevalence of metabolic disorders. These surveys obtained height, waist circumference (WC), and weight measurements for Chinese adults aged between 20 and 74 years who lived in urban communities. From the 1998–2001 survey, 4,894 participants (2,081 men and 2,813 women, mean age: 48.9 years) were recruited, and 4,395 participants (1,599 men and 2,796 women, mean age: 49.8 years) were recruited from the 2007–2008 survey. Using the World Health Organization criteria, overweight was defined as 25 kg/m^2^ ≤ BMI < 30 kg/m^2^ and obesity as BMI ≥ 30 kg/m^2^. Central obesity was defined as WC ≥ 90 cm in men or ≥85 cm in women. The differences in prevalence of obesity, central obesity and overweight between the two surveys were tested using multivariable logistic regression analyses.

**Results:**

Compared to the 1998–2001 survey, in the 2007–2008 survey the BMI distribution for men and the WC distribution for both genders is shifted significantly to the right along the *x*-axis (all p < 0.001). Over the ten years, the prevalence of combined overweight and obesity increased 24% (from 31.5% to 39.1%, p < 0.001) in men, but decreased 8% (from 27.3% to 25.0%; p < 0.01) in women. The prevalence of central obesity increased 40% in men (from 19.5% to 27.3%; p < 0.01), but the increase was not significant in women (15.0% to 17.1%; p *=* 0.051). In the total population, only central obesity showed a significant change between the populations in the two surveys, increasing 29% (from 17.3% to 22.4%; p < 0.001).

**Conclusions:**

Over this 10 year period, central obesity increased significantly in the Shanghai adult population. However, the prevalence of combined overweight and obesity was significantly increased in men but not in women.

## Background

In recent years, the prevalence of obesity in China has reached epidemic proportions, following rapid economic development and urbanization [1-3]. As obesity is an important and fundamental risk factor for cardiometabolic disorders, cardiovascular diseases and premature mortality [4-6], depicting the changing trends in obesity helps comprehend the prevalence of obesity-related chronic diseases and allows us to alert and inform the care professionals and the public in an attempt to prevent the epidemic. Body mass index (BMI) and waist circumference (WC) measurements are two important and practical indices that are used to identify general or central obesity, both in clinical practice and epidemiologic research. In general, central obesity is more closely associated with diabetes, whereas general obesity is more closely related with hypertension [5,7-10]. In addition, it has been reported that in some populations the mean WC is increasing more rapidly than mean BMI, especially in women, despite the fact that general obesity rates are stable or declining [11-15].

Shanghai is the largest city in southern China, and has recently experienced the most rapid economic development of mainland China. In Shanghai, we carried out two representative population-based cross-sectional surveys at an interval of approximately 10 years. These surveys, the 1998–2001 survey and the 2007–2008 survey, were designed to investigate the prevalence of metabolic disorders, and their protocols required the heights, weights, and waist circumferences of Chinese adults in Shanghai to be measured, allowing comparisons to be made in the changes in obesity measurements in this urban population over time. In the last decade, there have been no other comprehensive reports on the changing trends in the population prevalence of overweight, obesity or central obesity in urban Shanghai adults.

This study explores the secular changes in the prevalence of overweight, obesity and central obesity in Chinese adults in urban Shanghai by comparing two cross-sectional surveys that were carried out at an interval of ten years.

## Methods

This study was approved by the institutional review board of Shanghai Jiao Tong University Affiliated Sixth People’s Hospital, in accordance with the principles of the Helsinki Declaration II. Written informed consents were obtained from each participant.

### Study population

We analyzed datasets taken from two cross-sectional surveys with multistage sampling schemes carried out in Shanghai. The two surveys were designed to investigate the prevalence of metabolic disorders among Chinese adults. In 1998–2001, the Shanghai Diabetes Study (SHDS) examined a total of 5994 participants aged 15 to 95 years old from two urban communities in Shanghai, Huyang and Caoyang [16]. In 2007–2008, SHDS II surveyed a total of 5289 participants aged 20 to 74 years from six communities: four urban communities, Huayang, Linfeng, Tianmu, and Pengpu; one suburban community, Gongye; and one rural community, Anting [17]. The response rates were approximately 95% for both surveys [16,17]. These two survey populations did not overlap, as, in general, they involved different communities; the Huayang community was included in both studies, but different residential areas were investigated in each study. The following criteria were required for inclusion in the study: age 20–74 years; living in an urban or suburban community; complete data available for both WC and BMI. From the 1998–2001 survey, 4,894 participants (2,081 men and 2,813 non-pregnant women, mean age: 48.9 years) were selected for analysis, and 4,395 participants (1,599 men and 2,796 non-pregnant women, mean age: 49.8 years) were selected from the 2007–2008 survey. Both these two study populations have been described in our previous papers [16,17].

### Laboratory assessment of blood samples

As described previously, after 10 hours of overnight fasting, venous blood samples were drawn from participants without self-reported diabetes at 0 and 120 minutes after ingestion of a 75 g oral glucose load. Plasma glucose levels were measured using the glucose oxidase method [16,17]. Serum cholesterol and triglyceride levels were determined using different enzymatic assays in each of the two studies [16,17]. All laboratory measurements complied with a standardized and certified program; the details have been described previously [16,17].

### Physical examinations

Blood pressure, body weight, and height were measured according to standard protocols [18]. Body weight, height and WC were measured once for each survey. Height, WC, and weight were measured while the participants were barefoot and in light clothing using the Height & Weight Scale to the nearest 0.1 cm and 0.1 kg, respectively. BMI was calculated as weight divided by height squared (kg/m^2^). WC was measured at the horizontal plane between the inferior costal margin and the iliac crest on the mid-axillary line.

Demographic data on lifestyle, disease history, and family history of disease, smoking and drinking habits were collected during the survey using standardized questionnaires. The family history of obesity obtained information about obesity in first-degree relatives (biological mother, father, brothers, or sisters). Current smokers were defined as those who had smoked ≥ 1 cigarette/day for at least 1 year. Current drinkers were defined as those who had consumed ≥ 30 g of alcohol/week on average for at least 1 year. The educational level of participants was also recorded and categorized into three groups: low (illiterate, or having only attended primary and secondary education); medium (high school educated) and high (college or university educated). Monthly household income was classified as low level (< 1000 yuan RMB), medium level (1000–3000 yuan RMB), or high level (≥3000 yuan RMB).

### Definitions of obesity

Overweight and Obesity Two standards were used to diagnose overweight and obesity. Overweight is defined as BMI ≥ 25 kg/m^2^ and < 30 kg/m^2^ and obesity as BMI ≥ 30 kg/m^2^ using the World Health Organization (WHO) standards [19]. Using the Working Group on Obesity in China (WGOC) criteria, overweight is defined as a BMI between 24 kg/m^2^ and 28 kg/m^2^ and obesity as BMI ≥ 28 kg/m^2^[20].

Central Obesity Central obesity is WC ≥ 90 cm in men and ≥ 85 cm in women, as defined by the Chinese Joint Committee for Developing Chinese Guidelines on Prevention and Treatment of Dyslipidemia in Adults (JCDCG) [21].

### Statistical analysis

Descriptive statistics are presented as the mean (± standard deviation) or frequency (percentage). Age-adjusted p values for differences between means were calculated using covariance analyses (except for the non-adjusted p-value for age mean), or using multivariable logistic regression analyses for variables expressed as proportions (Table 1).

**Table 1:** Characteristics of Shanghai urban Chinese adults aged 20–74 years in 1998–2001 and 2007–2008

		**Men**			**Women**	
**Characteristic**	**1998-2001**	**2007-2008**	**p value**	**1998-2001**	**2007-2008**	**p value**
	**(N = 2081)**	**(N = 1599)**		**(N = 2813)**	**(N = 2796)**	
**Mean (SD)**						
Age (years)	48.9 (15.1)	50.1 (14.3)	0.005	48.9 (14.1)	49.7 (12.6)	<0.001
Height (cm)	169.3 (6.5)	169.3 (6.4)	0.397	157.5 (6.2)	158.3 (5.8)	<0.001
BMI (kg/m^2^)	23.7 (3.4)	24.2 (3.3)	<0.001	23.8 (3.6)	23.7 (3.4)	0.148
Waist circumference (cm)	82.3 (9.9)	84.9 (9.3)	<0.001	76.9 (10.0)	78.2 (9.3)	<0.001
Waist-to-height ratio	0.49 (0.1)	0.50 (0.1)	<0.001	0.49 (0.1)	0.50 (0.1)	0.017
Waist-to-hip ratio	0.89 (0.07)	0.89 (0.06)	0.107	0.83 (0.08)	0.84 (0.13)	0.748
SBP (mmHg)	125.5 (18.4)	124.5 (15.9)	0.008	121.9 (19.8)	120.4 (16.7)	0.660
DBP (mmHg)	81.1 (10.9)	79.4 (10.0)	0.005	77.6 (10.3)	75.8 (9.4)	<0.001
TC (mmol/L)	4.88 (1.10)	4.55 (0.91)	<0.001	5.04 (1.18)	4.72 (0.98)	<0.001
TG (mmol/L)	1.99 (1.57)	1.91 (1.59)	<0.001	1.73 (1.12)	1.55 (1.20)	<0.001
HDL-C (mmol/L)	1.26 (0.28)	1.19 (0.29)	<0.001	1.34 (0.29)	1.39 (0.31)	<0.001
LDL-C (mmol/L)	3.34 (0.98)	2.94 (0.77)	<0.001	3.43 (1.07)	2.98 (0.81)	<0.001
FPG (mmol/L)	5.29 (1.51)	5.68 (1.76)	<0.001	5.25 (1.34)	5.47 (1.34)	<0.001
2hPG (mmol/L)^a^	5.43 (1.71)	6.08 (1.63)	<0.001	5.56 (1.5)	6.12(1.51)	<0.001
***n *** **(%)**						
Family history of obesity	241 (12.9)	262 (17.3)	<0.001	350 (13.9)	517 (19.1)	<0.001
Smoking status			0.376			0.003
Non-smoker	738 (39.4)	619 (38.7)		2436 (96.7)	2746 (98.2)	
Current smoker	986 (52.7)	837 (52.3)		75 (3)	44 (1.6)	
Ex-smoker	147 (7.9)	143 (8.9)		8 (0.3)	6 (0.2)	
Dinking status			<0.001			0.314
Non-drinker	1364 (72.7)	1050 (65.7)		2479 (98.2)	2728 (97.6)	
Current drinker	506 (27)	494 (30.9)		43 (1.7)	63 (2.3)	
Ex-drinker	5 (0.3)	55 (3.4)		3 (0.1)	5 (0.2)	
Education			0.046			0.021
Low	726 (38.8)	663 (42.4)		1409 (56.0)	1421 (51.9)	
Medium	685 (36.6)	555 (35.5)		815 (32.4)	932 (34.0)	
High	459 (24.5)	347 (22.2)		290 (11.5)	387 (14.1)	
Income levels			<0.001			<0.001
Low	332 (17.7)	198 (13.2)		499 (19.8)	393 (15.1)	
Medium	1164 (62.2)	652 (43.5)		1644 (65.1)	1195 (45.9)	
High	375 (20.0)	648 (43.3)		382 (15.1)	1014 (39.0)	

Data are expressed as means (standard deviations) or as frequencies (%).

p-values (adjusted for age) for differences between means were calculated using a covariance analysis (ANCOVA, apart from the non-adjusted p-value for age), or using multivariable logistic regression analyses with variables shown as proportions.

^a^2 hPG was assessed in participants not known to have diabetes.

*Abbreviations*: BMI: body mass index; DBP: diastolic blood pressure; FPG: fasting plasma glucose; HDL-C: high density lipoprotein cholesterol; LDL-C: low density lipoprotein cholesterol; SBP: systolic blood pressure; TC: total cholesterol; TG: triglyceride; 2 hPG: 2 h post-load plasma glucose.

**Figure 1:**
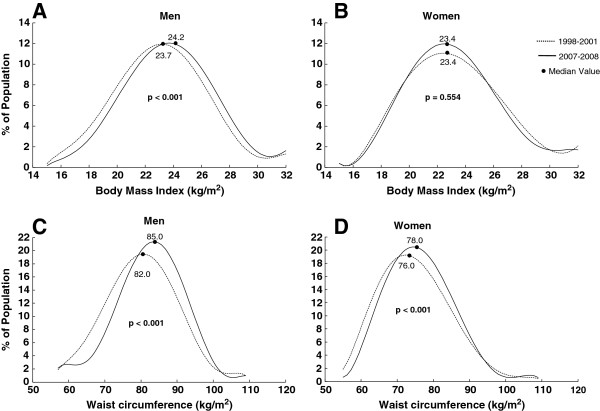
**Smoothed frequency distribution of body mass index and waist circumference for men and women from the two surveys.** The population percentage, on the *y*-axis, is plotted against BMI (Figure 1. **A-B**) or WC (Figure 1. **C-D**), on the *x*-axis. Curve fitting and loess smoothing were performed using Matalab2007a. The median figures are presented. The differences between the two surveys were tested with the Mann–Whitney U-test.

Standardized means and percentages were calculated using the direct method according to the Chinese population structure in 2000 [22] (Table 2, Figure 1, and Additional file 1: Table S1). Potential differences in age-adjusted means between the two surveys in both men and women were tested using covariance analyses (univariate general linear model, age-adjusted). Linear trends for age-specific means were tested using linear regression analyses, with age group being treated as a continuous variable (Table 2).

**Table 2:** Standardized means of body mass index and waist circumference in 1998–2001 and 2007–2008

**Population**	**N**_**1**_	**N**_**2**_	**BMI (kg/m**^**2**^**)**			**Waist circumference (cm)**		
	**1998-2001**	**2007-2008**	**1998-2001**	**2007-2008**	**Difference**^**e**^	**1998-2001**	**2007-2008**	**Difference**^**e**^
Standardized								
Overall^a^	4894	4395	23.3	23.6	0.2	77.7	79.9	1.4
Men^b^	2081	1599	23.5	24.1	0.5	81.1	83.9	2.6
Women^b^	2813	2796	23.0	23.1	−0.1	74.1	75.7	1.3
p values for difference^c^			0.257	p < 0.001		p < 0.001	p < 0.001	
Sex- and age-specific								
Men								
20-29 years	225	199	23.3 (4.2)	23.3 (3.8)	0	78.3 (11.1)	80.2 (10.7)	1.9
30-39 years	406	235	23.2 (3.3)	24.3 (3.5)	1.1	80.4 (9.3)	83.5 (8.9)	3.1
40-49 years	522	282	23.3 (3.1)	24.7 (3.1)	1.4	81.7 (9.4)	86.6 (8.7)	4.9
50-59 years	284	457	24.1 (3.1)	24.4 (3.2)	0.3	83.9 (9.5)	85.8 (8.8)	1.9
60-74 years	644	426	24.2 (3.2)	24.2 (3.0)	0	84.8 (9.5)	85.8 (9.0)	1
p values for linear trend^d^			p < 0.001	0.058		p < 0.001	p < 0.001	
Women								
20-29 years	225	249	21.2 (3.0)	21.6 (3.1)	0.4	67.6 (7.7)	70.8 (7.8)	3.2
30-39 years	561	427	22.8 (3.4)	22.8 (3.3)	0	73.0 (8.4)	73.8 (8.3)	0.8
40-49 years	841	590	23.5 (3.2)	23.8 (3.2)	0.3	75.5 (8.3)	78.2 (8.5)	2.7
50-59 years	385	968	24.5 (3.5)	24.1(3.4)	−0.4	79.0 (9.2)	79.8 (8.7)	0.8
60-74 years	801	562	25.2 (3.7)	24.5 (3.5)	−0.7	82.8 (9.9)	82.0 (9.0)	−0.8
p values for linear trend^d^			p < 0.001	p < 0.001		p < 0.001	p < 0.001	

Data are expressed as the means (with standard deviations).

Means were standardized by the direct method according to the Chinese population structure in 2000.

^a^Adjusted for age and sex.

^b^Adjusted for age.

^c^Differences in means were tested using an age-adjusted covariance analysis.

^d^Linear trends for age-specific means were tested using linear regression analyses; age group was treated as a continuous variable.

^e^The differences are calculated as the mean for the 2007–2008 survey minus the mean for the 1998–2001 survey.

*Abbreviations*: BMI: body mass index.

Potential differences in standardized proportions (prevalence) between the two surveys were tested using multivariable logistic regression analyses in men or women (adjusted for age), and among all participants (adjusted for sex and age), using the Entry method. Adjusted odds ratios (AORs) and p values were then calculated (Figure 1).

In addition, linear trends for proportions were tested using logistic regression analyses; again, age group was treated as a continuous variable. Differences in proportions (age-adjusted) between the two surveys in both men and women were tested using an age-adjusted multivariable logistic regression analysis (Additional file 1: Table S1).

All statistical analyses were performed using SPSS version 15.0 (SPSS Inc., Chicago, IL, USA). p < 0.05 (two-tailed) was designated as statistically significant. Curve fitting and lowess smoothing were performed using Matalab2007a.

The frequency distribution of BMI and waist circumference from the two surveys were smoothed using curve fitting with Matalab2007a, which is to construct a polynomial P(X) of degree N that fits the data Y best in a least-squares sense. The differences in the frequency distribution from the two surveys were tested with the Mann–Whitney *U*-test.

## Results

### Characteristics of Chinese adults in urban Shanghai aged 20–74 years in the 1998–2001 survey and in the 2007–2008 survey

The demographic and clinical characteristics of the men and women of both surveys are presented in Table 1. The mean WCs and mean waist-to-height ratios (a measure of waist circumference relative to height) were both higher in men and women in the 2007–2008 survey, as compared with the 1998–2001 survey, but there was no between-survey change in waist-to-hip ratios in either men or women. In men, the mean BMI was higher in the 2007–2008 survey, as compared to the 1998–2001 survey: 24.2 kg/m^2^ vs. 23.7 kg/m^2^, (p < 0.001). Both genders had significantly higher mean fasting plasma glucose levels and 2 h post-load glucose levels (2 hPG) in the 2007–2008 survey (p < 0.001); however, mean diastolic blood pressures (DBP) and mean lipid levels, apart from a decreased HDL-C level in men, were improved in the 2007–2008 survey (p < 0.01) (Table 1).

In the 2007–2008 survey, the participants noted a significant increase in the family history of obesity than in the 1998–2001 survey. Between the two surveys, there were favorable changes in smoking habits for women and adverse changes in drinking habits for men. Although improvements in income were observed in both genders, improvement in education was only apparent in women.

### BMI and WC frequency distributions in the 1998–2001 and 2007–2008 surveys

Figure 1 shows the frequency distributions of BMI and WC for men and women in 1998–2001 and in 2007–2008. Compared to the 1998–2001 survey, in the 2007–2008 survey the distribution of BMI for men is shifted significantly to the right along the *x*-axis, and the WC distribution is shifted to the right for both genders (all p < 0.001). There was no significant increase in median BMI in women (23.4 kg/m^2^ to 23.4 kg/m^2^, p = 0.554).

### BMIs and WCs in the 1998–2001 and 2007–2008 surveys

Table 2 shows significant increases in mean BMI (0.5 kg/m^2^, p < 0.001) for men, and for mean WC in both genders (2.6 cm for men and 1.3 cm for women, both p < 0.001) over the ten-year period. The standardized mean BMI in men increased from 23.5 kg/m^2^ in the 1998–2001 survey to 24.1 kg/m^2^ in the 2007–2008 survey (p < 0.001), but it was not significantly different between the women in these two populations (23.1 kg/m^2^ in 2007–2008 vs. 23.0 kg/m^2^ in 1998–2001; p = 0.148). In addition, the standardized mean WC was 77.7 cm in the 1998–2001 survey vs. 79.9 cm in the 2007–2008 survey (p < 0.001); 81.1 cm vs. 83.9 cm in men (p < 0.001) and 74.1 cm vs. 75.7 cm in women (p < 0.001).

### Prevalence of overweight, obesity, and central obesity in Chinese adults in the 1998–2001 and 2007–2008 surveys

**Figure 2:**
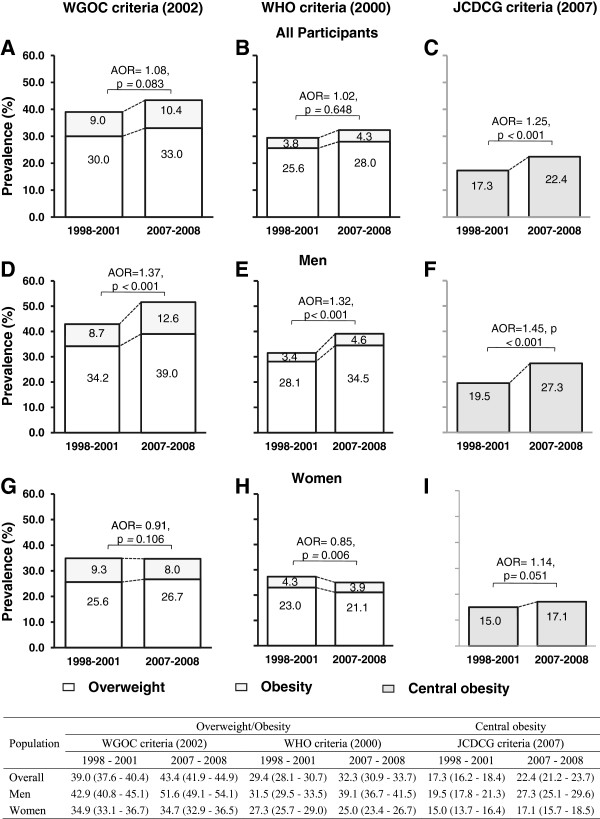
**Standardized prevalence of overweight, obesity and central obesity in urban Shanghai adults aged 20–74 years in the 1998–2001 and 2007–2008 surveys.** Standardized prevalence was calculated by the direct method according to the Chinese population structure in 2000 (Figure 2. **A-C** for all participants, Figure 2. **D-F** for men and Figure 2. **G-I** for women). Differences in standardized proportions (prevalence) between the two time periods were tested using multivariable logistic regression analyses in men or women (adjusted for age), and among total participants (adjusted for sex and age) using the Entry method. Adjusted odds ratios (AORs) and p values were then calculated. Standardized combined prevalence of overweight and obesity and prevalence of central obesity and their 95% CIs in urban Shanghai adults aged 20–74 years in the 1998–2001 and 2007–2008 surveys.

For the total population, only central obesity showed a significant increase in the 2007–2008 survey compared to the 1998–2001 survey (29% increase from 17.3% to 22.4%, p < 0.001; Figure 2). This increase was larger than the increase in prevalence of combined overweight and obesity between the two surveys. The increase in prevalence of combined overweight and obesity, of approximately 11% using the WGOC criteria (from 39.0% to 43.4%) or approximately 10% using the WHO criteria (from 29.4 to 32.3), did not reach statistical significance.

### Prevalence of overweight, obesity, and central obesity in men and women in the 1998–2001 and 2007–2008 surveys

Figure 2 shows the secular changes in prevalence of overweight, obesity, and central obesity over the 10-year period. Between the 1998–2001 and 2007–2008 surveys, using the WHO criteria, the prevalence of combined overweight and obesity increased by 24% (from 31.5% to 39.1%, p < 0.001; Figure 2) in men, but decreased by 8% (from 27.3% to 25.0%, p < 0.01) in women. Using the WGOC criteria, the prevalence of combined overweight and obesity increased in men, but were not significantly different between the two surveys in women. The prevalence of central obesity increased significantly by 40% in men (from 19.5% to 27.3%, p < 0.01; Figure 2), but the increase was non-significant in women (15.0% to 17.1%, p *=* 0.051).

The age-specific prevalence of central obesity, overweight, and obesity in men and women in the two surveys are shown in Additional file 1: Table S1.

## Discussion

In 1998–2001 and in 2007–2008, we carried out two independent population-based cross-sectional surveys with similar study protocols, involving the measurement of height, WC, and weight of Chinese adults, to investigate the prevalence of metabolic disorders in Shanghai. Here, WHO criteria were used for comparisons of our two surveys, and WCOG criteria were used for comparisons made between our surveys and the other nationwide Chinese surveys at the approximately same periods.

### Comparison of the results of our surveys with two other nationwide surveys of urban Chinese adults

During the time period covered by our surveys, there have been two other large nationwide population-based surveys carried out in China. In 2002, the China Health and Nutrition survey examined Chinese adults in urban areas aged ≥18 years [23], and in 2007–2008 the China National Diabetes and Metabolic Disorders Study [24] was conducted in Chinese adults aged ≥ 20 years in urban areas. The nationwide prevalence of combined overweight and obesity was 37.9% in 2002 [23] (vs. 39.0% in our study) increasing to 47.6% in 2007–2008 (vs. 43.4% in our study) [24], using WGOC criteria. The similar trends of increased prevalence of combined overweight and obesity were observed both nationwide and in urban Shanghai. However, the prevalence of combined overweight and obesity was lower in 2007–2008 in our Shanghai population than that was reported in the 2007–2008 China National Diabetes and Metabolic Disorders Study: 43.4% vs. 47.6%. In addition, the results from these two 2007–2008 reports showed that the prevalence of central obesity in the urban Shanghai population was also lower than that reported for urban Chinese adults: 22.4% vs. 30.0% [24].

Therefore, although the prevalence of central obesity in Shanghai urban population had increased over the period between our two studies, the prevalence of central obesity, and the prevalence of combined overweight and obesity is still lower than the national average at the same period.

### Gender differences in the prevalence of overweight, obesity, and central obesity over the 10-year period

There were obvious gender differences in the changing trends in the prevalence of combined overweight and obesity between our two studies. Between 1998–2001 and 2007–2008, the prevalence of combined overweight and obesity increased by 24% in men but decreased by 8% in women according to the WHO criteria. The prevalence of central obesity also showed a significant 40% increase in men, but there was non-significant increase in the prevalence of central obesity in women between the two studies.

Recent reports have shown that the prevalence of overweight, obesity, and central obesity among Chinese adults of both genders have increased greatly in urban and rural areas between 1993 and 2009 [2]. Our results indicate that the secular change in the prevalence of central obesity in Shanghai, the largest city in one of the most economically developed areas of southern China, was similar to that reported for the Hong Kong Chinese population between 1996 and 2005 [9]. This investigation showed that, although the prevalence of general obesity was steady in men but decreased in women, for central obesity, the prevalence increased in Hong Kong Chinese men and stay stable in Chinese women over a 10 year period [9]. Similar secular trends in the prevalence of obesity and central obesity have been observed among US adults [10-12].

These gender differences in obesity prevalence could be partly explained by increased exposure to drinking for men, and by the significant improvement in education level achieved by the women between the two surveys. This inverse association between education and obesity, which is found only in women, is described in our previous paper [25].

In addition, slower increases in the prevalence of overweight plus obesity were observed for men and women in urban Shanghai (42.9 to 51.6 and 34.9 to 34.7) compared with the national figures for urban Chinese men and women (41.4 to 55.0 and 35.3 to 40.2) during the same period according to the WGOC criteria [23,24].

### Metabolic disorders in the 1998–2001 and 2007–2008 surveys

Changes in other metabolic disorders over the 10-year period were also observed. In both men and women, after adjustment for age, there was an increase in mean fasting and 2-h glucose levels. However, mean blood pressure decreased and the lipid profiles of the population improved, with the exception of a decreased mean HDL-C level in men. Similar changes (decreased mean LDL-C levels, decreased systolic BP, and increased in HDL-C between surveys carried out in 1990 and 2001–2003) have been reported in a Hong Kong Chinese population [26].

### Advantages and limitations

The 1998–2001 survey and the 2007–2008 survey were elaborately designed to investigate the prevalence of metabolic disorders in Shanghai using representative study sample.

Different districts were sampled in the two surveys. In the 1998–2001 survey, both districts sampled were in urban areas; in the 2007–2008 survey, four of the sampled districts were in urban areas and one district was in a suburban area. This complicates the analysis of the changes in prevalence of overweight and obesity. The changing trends in lipidemia over the 10-year period were not fully explored due to the differences in the laboratory analysis methods used in the 1998–2001 and 2007–2008 surveys.

## Conclusions

Over the 10-year period covered by our surveys, there was a larger observed increase in the prevalence of central obesity than in the prevalence of combined overweight plus obesity in this urban Shanghai population. In comparison with their nationwide counterparts, this Shanghai population showed a slower growth in the prevalence of combined overweight and obesity. Significant increases in the prevalence of combined overweight and obesity, and of central obesity, were observed in men, but there was a stable or declining prevalence of combined overweight and obesity in women. Multiple community-based, gender-specific strategies are urgently required to combat the increasing prevalence of overweight and obesity in the Shanghai area.

## Competing interests

The authors declare that no competing interest exists.

## Authors’ contribution

XH performed the statistical analysis and wrote the manuscript; YL and HL participated in the data collection and checked the data; XM and CH contributed to discussion; WJ and YB participated in the design of this study and edited the manuscript. All authors have read and approved the final manuscript.

## Pre-publication history

The pre-publication history for this paper can be accessed here:

http://www.biomedcentral.com/1471-2458/13/1064/prepub

## Supplementary Material

Additional file 1: Table S1 Standardized prevalence of overweight, obesity and central obesity in 1998–2001 and 2007–2008. Click here for file
