# Supplementary material for: Ten-year changes in the prevalence of overweight, obesity and central obesity among the Chinese adults in urban Shanghai, 1998–2007 — comparison of two cross-sectional surveys
Source: BMC Public Health. 2013 Nov 12;13:1064. doi: 10.1186/1471-2458-13-1064 (PMC3833854; doi:10.1186/1471-2458-13-1064)
Supplement: Additional file 1: Table S1 — Standardized prevalence of overweight, obesity and central obesity in 1998–2001 and 2007–2008. [file 1471-2458-13-1064-S1.doc]

Additional file 1: Table S1. Standardized Prevalence of Overweight , Obesity and Central Obesity in 1998-2001 and 2007-2008

| **Population** | **1998-2001** | | | | | **2007-2008** | | | | |
| --- | --- | --- | --- | --- | --- | --- | --- | --- | --- | --- |
|  | WHO Standard | | WGOC Standard | | Central  Obesity | WHO Standard | | WGOC Standard | | Central  Obesity |
|  | Overweight | Obesity | Overweight | Obesity |  | Overweight | Obesity | Overweight | Obesity |  |
| Standardized |  |  |  |  |  |  |  |  |  |  |
| Overall^a^ | 25.6 | 3.8 | 30.0 | 9.0 | 17.3 | 28.0 | 4.3 | 33.0 | 10.4 | 22.4 |
| Men^b^ | 28.1 | 3.4 | 34.2 | 8.7 | 19.5 | 34.5 | 4.6 | 39.0 | 12.6 | 27.3 |
| Women^b^ | 23.0 | 4.3 | 25.6 | 9.3 | 15.0 | 21.1 | 3.9 | 26.7 | 8.0 | 17.1 |
| *P* values^c^ | 0.533 | *P* < 0.001 | *P* = 0.001 | *P* < 0.001 | 0.238 | *P* < 0.001 | 0.501 | *P* < 0.001 | 0.169 | *P* < 0.001 |
| Sex- and age-specific |  |  |  |  |  |  |  |  |  |  |
| Men |  |  |  |  |  |  |  |  |  |  |
| 20-29 | 56 (24.9) | 11 (4.9) | 65 (28.9) | 24 (10.7) | 30 (13.3) | 50 (25.1) | 10 (5.0) | 56 (28.1) | 26 (13.1) | 37 (18.6) |
| 30-39 | 105 (25.9) | 9 (2.2) | 133 (32.8) | 30 (7.4) | 68 (16.7) | 88 (37.4) | 10 (4.3) | 95 (40.4) | 31 (13.2) | 54 (23.0) |
| 40-49 | 132 (25.3) | 13 (2.5) | 177 (33.9) | 31 (5.9) | 101 (19.3) | 114 (40.4) | 15 (5.3) | 129 (45.7) | 35 (12.4) | 105 (37.2) |
| 50-59 | 101 (35.6) | 11 (3.9) | 118 (41.5) | 29 (10.2) | 77 (27.1) | 160 (35.0) | 24 (5.3) | 186 (40.7) | 62 (13.6) | 150 (32.8) |
| 60-74 | 233 (36.2) | 24 (3.7) | 264 (41.0) | 67 (10.4) | 201 (31.2) | 157 (36.9) | 12 (2.8) | 193 (45.3) | 39 (9.2) | 142 (33.3) |
| *P* values for trend^d^ | *P* < 0.001 | 0.679 | *P* < 0.001 | 0.198 | *P* < 0.001 | 0.078 | 0.280 | *P* < 0.001 | 0.162 | *P* < 0.001 |
| Women |  |  |  |  |  |  |  |  |  |  |
| 20-29 | 24 (10.7) | 4 (1.8) | 29 (12.9) | 6 (2.7) | 9 (4.0) | 26 (10.4) | 5 (2.0) | 43 (17.3) | 7 (2.8) | 14 (5.6) |
| 30-39 | 101 (18.0) | 24 (4.3) | 119 (21.2) | 47 (8.4) | 52 (9.3) | 69 (16.2) | 15 (3.5) | 90 (21.1) | 28 (6.6) | 48 (11.2) |
| 40-49 | 226 (26.9) | 28 (3.3) | 256 (30.4) | 73 (8.7) | 120 (14.3) | 162 (27.5) | 22 (3.7) | 200 (33.9) | 54 (9.2) | 127 (21.5) |
| 50-59 | 128 (33.2) | 26 (6.8) | 135 (35.1) | 64 (16.6) | 100 (26.0) | 308 (31.8) | 57 (5.9) | 339 (35.0) | 123 (12.7) | 263 (27.2) |
| 60-74 | 345 (43.1) | 70 (8.7) | 355 (44.3) | 144 (18.0) | 320 (40.0) | 186 (33.1) | 37 (6.6) | 215 (38.3) | 85 (15.1) | 208 (37.0) |
| *P* values for trend^d^ | *P* < 0.001 | *P* < 0.001 | *P* < 0.001 | *P* < 0.001 | *P* < 0.001 | *P* < 0.001 | *P* < 0.001 | *P* < 0.001 | *P* < 0.001 | *P* < 0.001 |

Data are expressed as the frequency (%). Proportions were standardized by the direct method according to the Chinese population structure in 2000.

^a^Adjusted for age and sex.

^b^Adjusted for age.

^c^Differences in age-adjusted proportions between men and women were tested using multivariable logistic regression analysis (adjusted for age).

^d^Linear trends for sex- and age-specific proportions were tested using logistic regression analyses, here, age group was treated as a continuous variable.

WHO standard: overweight, 25 kg/m^2^ ≤ BMI < 30 kg/m^2^; obesity, BMI ≥ 30 kg/m^2^.

WGOC standard: overweight, 24 kg/m^2^ ≤ BMI < 28kg/m^2^; obesity, BMI ≥ 28 kg/m^2^.

Central obesity: waist circumference ≥ 90 cm in men and ≥ 85 cm in women.
